# An Analysis of Emergency Surgical Outcomes for Pediatric Traumatic Brain Injury: A Ten-Year Single-Institute Retrospective Study in Taiwan

**DOI:** 10.3390/medicina60091518

**Published:** 2024-09-18

**Authors:** Cheng-Yu Tsai, Keng-Liang Kuo, Chieh-Hsin Wu, Tai-Hsin Tsai, Hui-Yuan Su, Chih-Lung Lin, Ann-Shung Lieu, Aij-Lie Kwan, Yu-Feng Su, Joon-Khim Loh

**Affiliations:** 1 Division of Neurosurgery, Department of Surgery, Kaohsiung Medical University Hospital, Kaohsiung 807, Taiwan; chengyutsai@kmu.edu.tw (C.-Y.T.); u9101089@gmail.com (K.-L.K.); wujoeys@gmail.com (C.-H.W.); teishin8@hotmail.com (T.-H.T.); latimeria8@hotmail.com (H.-Y.S.); chihlung1@yahoo.com (C.-L.L.); e791125@gamil.com (A.-S.L.); aijliekwan@yahoo.com.tw (A.-L.K.); 2 Department of Post-Baccalaureate Medicine, Kaohsiung Medical University, Kaohsiung 807, Taiwan; 3 Graduate Institute of Medicine, College of Medicine, Kaohsiung Medical University, Kaohsiung 807, Taiwan; 4 Division of Neurosurgery, Department of Surgery, Kaohsiung Medical University Gangshan Hospital, Kaohsiung 807, Taiwan

**Keywords:** pediatric brain injury, surgical outcome, decompressive craniectomy, post-traumatic hydrocephalus

## Abstract

*Background and Objectives*: Pediatric traumatic brain injury (pTBI) remains a major pediatric public health problem, despite well-developed injury prevention programs. The purpose of this study is to analyze the emergency surgical outcomes of pTBI in a single institute ten-year retrospective study to offer a real-world clinical result. *Materials and Methods*: Our institute presented a clinical retrospective, single-institute research study of 150 pediatric TBI cases that were diagnosed and underwent emergency surgical treatment from 2010 to 2019. *Results*: The incidence of radiological findings is detailed as follows: brain edema (30%, 45/150), followed by acute subdural hematoma (27.3%, 41/150), epidural hematoma (21.3%, 32/150), chronic subdural hemorrhage (10%, 15/150), skull fracture (6.7%, 10/150), and traumatic subarachnoid hemorrhage (4.7%, 7/150). Surgical intervention data revealed that decompressive craniectomy was still the main effective surgical method. The results showed longer hospital stays and higher morbidity rates in the brain edema, acute subdural hematoma, and chronic subdural hemorrhage groups, which were viewed as poor surgical outcome groups. Epidural hematoma, skull fracture and traumatic subarachnoid hemorrhage were categorized into good surgical outcome groups. Notably, the data revealed gross improvement in Glasgow Coma Scale/Score (GCS) evolution after surgical interventions, and the time to cranioplasty was a significant factor in the development of post-traumatic hydrocephalus (PTH). *Conclusions*: Our study provided real-world data for the distribution of etiology in pTBI and also categorized it into six groups, indicating disease-orientated treatment. In addition, our data supported that decompressive craniectomy (DC) remains a mainstay surgical treatment in pTBI and early cranioplasty could decrease the incidence of PTH.

## 1. Introduction

Traumatic brain injury (TBI) is still a leading cause of death and a health problem in the worldwide pediatric population [1,2]. As reported, the worldwide incidence of pediatric TBI (pTBI) ranges varies broadly by country, with most reporting a range between 47 and 280 per 100,000 children [3]. The pTBI was associated with various kinds of negative sequelae, such as cognitive deficits, behavioral problems, poor school performance, and declines in adaptive behavior. The causes of pTBI are varied and distributed by age. Traffic accidents (motor vehicle accidents, MVA) is the most common cause, followed by falls (11%), unknown causes (5%), and assaults (4%). However, sports-related pTBI increases in middle childhood (around 10 years of age) and pTBI from motor vehicle-related causes and assaults peaks in late adolescence [4].

In an effort to reduce the consequences of pTBI, increasing programs for injury prevention programs were developed. From prehospital management to intensive medical care, all are designed to improve the clinical outcomes of pTBI. Prehospital management plays a pivotal role in pTBI survival by stabilizing patients for transport, preventing secondary brain injuries, and triaging those with severe injuries to appropriate levels of care [5,6]. Furthermore, the management of severe TBI at dedicated pediatric trauma centers with a standardization of intensive care could improve clinical outcomes. The steps for the standardization of care are well established, such as diagnostic tools, the prevention of epilepsy agents, and surgical methods and indications. However, despite advancements in prevention programs and medical treatment, pTBI remains a major public health concern, posing a significant burden on the healthcare system. Therefore, we attempted to provide detailed insight into the emergency surgical aspects of our experience to improve the quality of pTBI.

In this study, we retrospectively analyzed our results of 150 emergency surgical pediatric TBI cases from a single institute over a 10-year period. Detailed demographic, clinical, imaging, histopathological features, and outcomes were studied. We aimed to analyze the various aspects of emergency surgical clinical outcomes and prognosis to enhance the understanding and experience of pTBI.

## 2. Materials and Methods

### 2.1. Data Collection

We retrospectively collected 150 consecutive pediatric patients under the age of 18 years who had a single head injury and underwent emergency surgery for TBI between 2010 and 2019 in our single institute for inclusive criteria. The retrospective study was approved by our hospital’s institutional review board/ethics committee, and informed consent from the patients was obtained. Recurrent cases of pTBI were excluded from the current study. The exclusion criteria were repeated head trauma and a combination of other organ trauma. The data were obtained from the hospital’s medical record section. The relevant demographics, clinical details, imaging features, surgical details, and outcomes were studied. Conventional brain computed tomography (CT) was carried out on all the patients. The decision to perform emergency surgery was made based on the clinical presentation and/or radiological progression of the disease. Surgical indications included removing space-occupying disorders, such as hematomas, contusions and depressed skull fragments, and maintaining a normal intracranial pressure (ICP) (usually <20 mmHg) and optimal cerebral perfusion pressure (CPP) (>40–50 mmHg) with cerebrospinal fluid (CSF) diversion. 

### 2.2. Radiological Assessment

**Figure 1:**
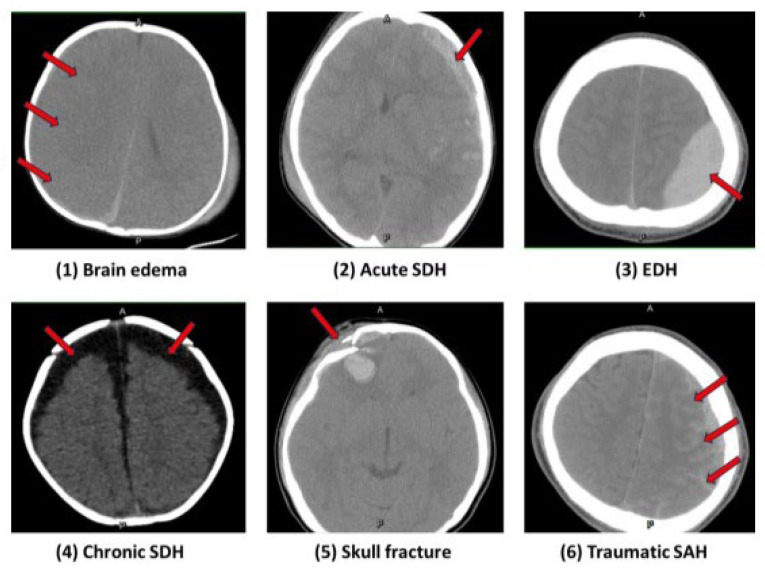
The six major groups are listed as follows: (**1**) brain edema, (**2**) acute subdural hematoma (acute SDH), (**3**) epidural hematoma (EDH), (**4**) chronic subdural hemorrhage (chronic SDH), (**5**) skull fracture, and (**6**) traumatic subarachnoid hemorrhage (traumatic SAH).

According to the brain CT image findings, the surgical groups were divided into six major groups as follows: (1) brain edema (midline shifting more than 0.5 cm) (2) acute subdural hematoma (acute SDH), (3) epidural hematoma (EDH), (4) chronic subdural hemorrhage (chronic SDH), (5) skull fracture, and (6) traumatic subarachnoid hemorrhage (traumatic SAH), as shown in Table 1 and Figure 1. All analyses and results were performed according to the six subgroups presented.

In Table 2 and Table 3, we also summarized baseline demographic and clinical variables in the entire patient population (total) and in the six groups, including basic parameters, cause of damage, surgical intervention, GCS evolution, and outcome analysis.

### 2.3. Surgical Interventions

Standard primary decompressive craniectomy (DC) or craniotomy was performed for (1) brain edema, (2) acute SDH, (3) EDH, (4) chronic SDH, and (5) skull fracture.

The decision to perform emergency surgery was made based on a clinical presentation (Glasgow Coma Scale/Score (GCS) below 8 or a descending GCS of more than 2 and/or the radiological progression of the disease (hematoma volume more than 20 mL or hematoma diameter more than 1 cm and midline shift more than 0.5 cm under CT findings for SDH, EDH, brain edema and chronic SDH). The standard surgical procedure of burr holes or craniotomy with evacuation was performed for (1) brain edema, (2) acute SDH, (3) EDH, (4) chronic SDH, and (5) skull fracture. The number of burr holes made was decided based on the imaging findings. The decision to keep the subdural drain was left to the surgeon’s discretion or the lack of brain surfacing at the end of surgery. The drain was removed within 48 h after obtaining a plain CT head.

Intracranial pressure monitoring (ICP monitor) was performed for suitable cases of (1) brain edema, (2) acute SDH, (3) EDH, (4) chronic SDH, and (5) skull fracture.

Extra-ventricular drainage (EVD) was performed at Kocher’s point for hydrocephalus or ICP monitoring in all six groups.

### 2.4. GCS (Glasgow Coma Scale/Score) Evolution

**Figure 2:**
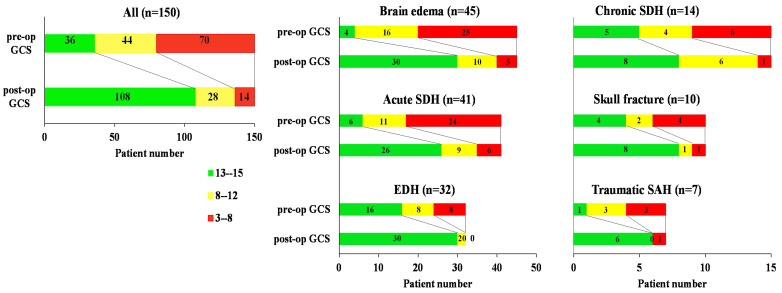
GCS (Glasgow Coma Scale/Score) Evolution. The evolution of the GCS before and after the operation was further investigated in six groups. An improvement in GCS was detected in each group (All, brain edema, acute SDH (subdural hematoma), EDH (epidural hematoma), chronic SDH (chronic subdural hemorrhage), skull fracture and traumatic SAH (subarachnoid hemorrhage).

We divided all patients into three groups based on pre-operation GCS (pre-op GCS) and post-operation GCS (post-op GCS): mild (GCS 13–15), moderate (GCS 9–12), and severe (GCS 3–8) in Figure 2.

### 2.5. Complications

We also analyzed complications, including mortality (death), respiratory failure, re-bleeding cases, and post-traumatic hydrocephalus (PTH). Notably, PTH was further analyzed and recorded as a risk factor in Table 4. In addition, data on hospital stays were also obtained in our study.

### 2.6. Statistical Analysis

All data are expressed as means and SDs for variables. For each group, we used an independent samples *t*-test, Fisher’s exact test, the Wilcoxon–Mann–Whitney test, one-way-ANOVA, and Kruskal–Wallis test, with statistical significance set at *p* < 0.05. All analyses were performed using the Statistical Package for the Social Science (version 17) (SPSS, International Business Machines Corporation Software, Armonk, New York, NY, USA).

## 3. Results

### 3.1. Imaging Findings

The most common radiological finding of pTBI patients was brain edema (30%, 45/150), followed by acute SDH (27.3%, 41/150), EDH (21.3%, 32/150), chronic SDH (10%, 15/150), skull fracture (6.7%, 10/150), and traumatic SAH (4.7%, 7/150), as shown in Table 1.

### 3.2. Basic and Demographic Profiles 

The mean age was 10.9 ± 7.4 years old, and chronic SDH was diagnosed at a relatively young age (1.8 ± 1.7 years old), with significance (*p*-value = 0.00049). Males predominated in each group, except chronic SDH (males were equal to females). MVA was the most common cause (62%, 93/150), followed by a fall (20%, 30/150), assault (11.3%, 17/150), and birth trauma (6.6%, 10/150). 

### 3.3. Surgical Interventions

Decompressive craniectomy was still a popular surgical method for brain edema and acute SDH (26/45 and 21/41). Craniotomy was the major surgical method for EDH (26/32). ICP monitoring was the most common surgical procedure in each group. In Table 3, however, the difference between the groups was not statistically significant.

### 3.4. GCS Evolution

The evolution of the GCS before and after the operation was further investigated, as depicted in Table 3 and Figure 2. We divided the patients into three groups based on GCS score: severe group (3–8), moderated group (9–12), and mild group (13–15). The data revealed that post-operation GCS (post-op GCS) improved in total and in each group. According to GCS evolution data, EDH, skull fracture, and traumatic SAH were categorized into good surgical outcome groups (>80% in the mild group), while brain edema, acute SDH, and chronic SDH were viewed as poor surgical outcome groups (>30% in the mild group).

### 3.5. Prognosis and Complications

The average hospital stay was 32.8 days, with acute SDH (36.4 days) and chronic SDH (58.8 days) being longer than the average days in Table 3. In the brain edema and acute SDH groups, there were 12 mortalities (deaths) (12/150, 8%). Respiratory failure and tracheotomy were the main complications in 18.6% (28/150), which was relatively high in the brain edema and acute SDH groups. Re-bleeding after the second operation was the second main complication (9.3%, 14/150), and it was slightly predominated in the brain edema group.

### 3.6. Post-Traumatic Hydrocephalus (PTH)

Finally, we focused on PTH and evaluated the risk factors, such as age and time to cranioplasty. The results revealed that time to cranioplasty was a risk factor for the incidence of PTH (yes vs. no = 171 days vs. 110 days), as shown in Table 4.

## 4. Discussion

In our study, we presented and analyzed the clinical emergency surgical outcomes and prognoses of pTBI. In reviewing the literature in English, our results provided a large-scale retrospective study of the surgical aspects of pTBI. Our data revealed the radiological distribution of pTBI, which included brain edema, acute SDH, EDH, chronic SDH, skull fracture, and traumatic SAH. We also categorized them into six groups for further analysis. MVA remained the most common cause of pTBI, followed by falls, assault, and birth trauma. Notably, assault was a relatively higher incidence due to the socioeconomic level in our region. In a review of the literature, patients who underwent DC had 22% lower mortality rates, albeit with higher rates of a vegetative state, lower severe disability, and upper severe disability compared to the medically managed cohort [7]. To date, there have been no dedicated randomized controlled trials evaluating DC in pediatric TBI. However, DC was still the mainstay procedure in pTBI and offered favorable surgical results in our study. However, PTH post-DC needs to be taken into consideration, and a short time to cranioplasty is appropriate. Pre-op and post-op GCS were the most important emergency surgical results in pTBI. Our data also revealed a significant improvement in GCS evolution after the operation, indicating that the surgical procedure is still the vital treatment in pTBI. Finally, according to our statistical results, brain edema, acute SDH, and chronic SDH were categorized into poor surgical outcome groups. Conversely, EDH, skull fracture and traumatic SAH were viewed as good surgical outcome groups.

### 4.1. Brain Edema

In general, brain edema is more prevalent in pTBI than in focal injuries and contusions in adult TBI. In our study, brain edema (intracranial hypertension) is the most frequent radiological finding in pTBI, whether global or focal, and peaks 24–72 h after injury. Brain edema exacerbates injury by limiting blood flow, substrate diffusion, and oxygen and glucose delivery [8]. One of the most vital concepts of TBI management is maintaining “appropriate” ICP and CPP (CPP = mean arterial pressure [MAP] − ICP). Initial adequate resuscitation and the correction of respiratory with circulatory failure are mandatory when treating patients with pTBI. Management strategies are standardized and include CSF diversion, sedation, analgesics, neuromuscular blockade, hyperosmolar therapy, blood pressure augmentation, mild hyperventilation, and decompressive craniectomy (DC). However, DC, with or without duraplasty, may be considered in the treatment of brain edema refractory to medical management or for patients at risk of cerebral herniation [9]. In our series, the incidence of brain edema receiving surgical treatment was 45/150 (30%) in all groups, and MVA was still the leading cause. Decompressive craniectomy remains a popular surgical method (26/45, 57.7%). GCS evolution (8.9% to 33.3%) improved after surgical treatment. Although mortality (11%) and respiratory and re-bleeding rates were the highest compared with the other groups, decompressive craniectomy is still the mainstay procedure for refractory intracranial hypertension in our study. Therefore, surgical therapy is still mainstay procedure for brain edema in pediatric population based on our data.

### 4.2. Acute SDH and EDH

As reported, EDH was relatively more common than acute SDH due to the relatively looser dural space in the pediatric population. In our series, acute SDH and EDH were the most popular radiological findings for pTBI, except for brain edema. Both were common clinical diseases with the same trauma cause, and most were motor vehicle accidents. However, based on our study, EDH had a better GCS evolution than acute SDH. 

In addition, shorter hospital days (13 days, EDH vs. 41.97 days, acute SDH) and low mortality (0%, EDH vs. 9.7%, acute SDH) in the EDH group were noted, and a low incidence of respiratory failure (3.1%, EDH vs. 26.8%, acute SDH) and re-bleeding rate (3.1%, EDH vs. 7.3%, acute SDH) were also recorded. Therefore, acute SDH still had poor outcomes and more profound effects than EDH in pediatric populations. According to our result, a surgical procedure is also the standard therapy for acute SDH and EDH in the pediatric population. 

### 4.3. Chronic SDH

Chronic subdural hemorrhage is a relatively uncommon disease in pediatric populations—only five major case series are present in the recent English literature [10,11,12,13,14]. Child abuse, birth trauma, coagulopathy, and shunt surgeries are the major causes in this group. The common symptoms/signs are headache, vomiting, seizure, focal neurological deficit, or altered sensorium. In infants, it could present as refusal to feed, increased head circumference, and the delayed achievement or regression of milestones. Surgery is the mainstay procedure, and repeat drainage was required in some cases. In our study, the mean patient age was 3.0 ± 6.2 years (range 1 day–17 years), with 5 males (33.33%) and 10 females (66.66%). Infants account for 11/15 (73.33%), while others account for 4/15 (26.66%; 6–17 years old). Child abuse was reported in our series (7/15). Long bones, eye fundus examinations, and skin bruises at different stages of healing are tiny clues for child abuse. Prolonged hospital stays (58.8 days) were also found, resulting in the acquisition of more intensive medical care and a life-support system. Therefore, a detailed metabolic and skeletal workup is required to rule out child abuse in cases where chronic SDH was initially diagnosed to improve care provider quality and the long-term social care system. In addition, the poor improvement of GCS evolution and relatively high mortality demonstrated that chronic SDH was a unique status in pTBI that required more intensive medical care. Based on our study, surgical treatment, such as burr hole drainage, is a reliable therapy for chronic SDH in the pediatric population.

### 4.4. Skull Fracture and Traumatic SAH

Skull fractures and traumatic SAH are special groups for pTBI. They were viewed as relatively minor trauma and good surgical outcome groups in our study. Short hospital stays (14.8 days and 17.8 days, respectively) and fewer complication rates (mostly less than 10%) were recorded. Our work revealed that ICP implantation and standard craniotomy/burr bole drainage were effective surgical methods for offering adequate medical treatment.

### 4.5. Post-Traumatic Hydrocephalus (PTH)

Finally, PTH in pediatric patients after decompressive craniectomy is still being debated. Studies have shown that the incidence in the pediatric population can range from 15% to 40% after DC [15]. Disrupted CSF dynamics (including hydrocephalus and hygromas), infection, seizures, and syndrome of the trephined were viewed as etiology. A longer time to cranioplasty was associated with an increased likelihood of PTH [16]. In our series, six patients with PTH (6/59.4%) were reported in addition to four cases of SDH, followed by one case of chronic SDH and one case of brain edema. The average number of days from DC to cranioplasty was 110.9 ± 31.5 days in the non-PTH group in comparison with 171.1 ± 39.7 days in the PTH group, with a significant difference (*p*-value = 0.005) in Table 4. Our findings are consistent with previous reports, and early cranioplasty could decrease the incidence of PTH in pediatric populations after DC. Moreover, acute SDH was a high incidence in our study due to the disruption of CSF dynamics.

## 5. Conclusions

Pediatric TBI is still a leading cause of morbidity and mortality in children worldwide, with complex interactions between injuries and emergency surgical outcomes. Our study provided the real-world data for the distribution of etiology in pTBI and also categorized it into six groups, indicating disease-orientated treatment. In addition, our data supported that DC remains a mainstay surgical treatment in pTBI. Finally, our results demonstrated that early cranioplasty could decrease the incidence of PTH. Understanding the pathophysiology and surgical strategies for pediatric TBI is the main cornerstone. Our work attempted to provide overall emergency surgical viewpoints for medical care, while also offering our experiences to improve the surgical outcome in pTBI.

## Figures and Tables

**Table 1:** Radiological profiles of 150 pediatric traumatic brain injury patients who underwent surgical treatment.

	Patient Number	%
Brain edema	45	30.0
Acute subdural hematoma	41	27.3
Epidural hematoma	32	21.3
Chronic subdural hemorrhage	15	10.0
Skull fracture	10	6.7
Traumatic subarachnoid hemorrhage	7	4.7

**Table 2:** Basic and demographic profiles of 150 pediatric traumatic brain injury patients who underwent surgical treatment.

	All (n = 150)	Brain Edema (n = 45)	Acute SDH (n = 41)	EDH (n = 32)	Chronic SDH (n = 15)	Skull Fracture (n = 10)	Traumatic SAH (n = 7)	*p*-Value
Age, years, mean (OR)	10.9 ± 7.4	13.1 ± 6.4	9.9 ± 7.4	12.1 ± 6.2	1.8 ± 1.7	12.2 ± 5.7	10.9 ± 8.4	0.00049 ^a^
Male–Female	98:52	33:12	28:13	20:12	5:10	7:3	5:2	0.129 ^b^
Cause								
Motor vehicle accident	93 (62%)	31 (68.8%)	24 (58.5%)	19 (59.3%)	4 (26.6%)	10 (100%)	5 (71.4%)	0.221 ^c^
Fall down	30 (20%)	9 (20%)	8 (19.5%)	8 (25%)	3 (20%)	0 (0%)	2 (28.6%)	
Assault	17 (11.3%)	5 (11.1%)	5 (12.1%)	3 (9.3%)	4 (26.6%)	0 (0%)	0 (0%)	
Birth trauma	10 (6.6%)	0 (0%)	4 (9.7%)	2 (6.2%)	4 (26.6%)	0 (0%)	0 (0%)	

^a^ Using ANOVA-one way test; ^b^ Pearson’s chi-squared test; ^c^ Kruskal-Wallis test. Acute SDH: acute subdural hematoma; EDH: epidural hematoma; chronic SDH: chronic subdural hemorrhage; traumatic SAH: traumatic subarachnoid hemorrhage.

**Table 3:** Clinical parameters and complications of 150 pediatric traumatic brain injury patients who underwent surgical treatment.

	All (n = 150)	Brain Edema (n = 45)	Acute SDH (n = 41)	EDH (n = 32)	Chronic SDH (n = 15)	Skull Fracture (n = 10)	Traumatic SAH (n = 7)	*p*-Value
Intervention								
craniectomy	59	26	21	5	2	5	0	
craniotomy/burr hole	61	8	13	26	9	5	0	0.185 ^c^
ICP monitor	95	33	32	19	4	7	0	
EVD	12	1	4	0	1	0	7	
Pre-op GCS								
3–8	70 (46.6%)	25 (55.5%)	24 (58.5%)	8 (25%)	6 (40%)	4 (40%)	3 (42.8%)	
9–12	44 (29.4%)	16 (35.5%)	11 (26.8%)	8 (25%)	4 (26.6%)	2 (20%)	3 (42.8%)	0.028 ^c^
13–15	36 (24%)	4 (8.9%)	6 (14.6%)	16 (50%)	5 (33.3%)	4 (40%)	1 (14.2%)	
Post-op GCS								
3–8	14 (9.3%)	5 (11.1%)	6 (14.6%)	0 (0%)	1 (6.6%)	1 (10%)	1 (14.3%)	
9–12	28 (18.6%)	10 (22.2%)	9 (22%)	2 (6.2%)	6 (40%)	1 (10%)	0 (0%)	0.014 ^c^
13–15	108 (72%)	30 (33.3%)	26 (63.4%)	30 (93.8%)	8 (53.4%)	8 (80%)	6 (85.7%)	
Outcome analysis								
Hospital days	32.8	36.4	41.97	13	58.8	14.8	17.8	
Mortality	12 (8%)	5 (11%)	4 (9.7%)	0 (0%)	1 (6%)	1 (10%)	1 (14%)	0.003 ^c^
Respiratory failure	28 (18.6%)	11 (24.5%)	11 (26.8%)	1 (3.1%)	4 (26.7%)	1 (10%)	0 (0%)	
Re-bleeding	14 (9.3%)	8 (17.8%)	3 (7.3%)	1 (3.1%)	1 (6.6%)	1 (10%)	0 (0%)	
PTH	6 (6/59; 10%)	1 (1/26; 3.84%)	4 (4/21; 19%)	0 (0%)	1 (1/2; 50%)	0 (0%)	0 (0%)	

^c^ Kruskal–Wallis test. Pre-op, pre-operation; post-op, post-operation; GCS, Glasgow Coma Scale/Score; PTH, post-traumatic hydrocephalus. Acute SDH: acute subdural hematoma; EDH: epidural hematoma; chronic SDH: chronic subdural hemorrhage; traumatic SAH: traumatic subarachnoid hemorrhage.

**Table 4:** Clinical parameters and risk factors of post-traumatic hydrocephalus after decompressive craniectomy.

Risk factor	Hydrocephalus				
	Yes		No		
	Number	%	Number	%	*p*-Value
Sex					
Male	4	9.3	39	90.69	0.390 ^a^
Female	2	12.5	14	87.5	
Image findings					
Brain edema	1	4	25	96	
Acute SDH	4	19	17	80.9	
EDH	0	0	5	100	0.392 ^b^
Chronic SDH	1	50	1	50	
Skull fracture	0	0	5	100	
Traumatic SAH	0	0	0	0	
Cranioplasty					
Yes	5	18.5	22	81.48	0.833 ^a^
No	1	3.1	31	96.87	
	Hydrocephalus				
Risk factor	Yes		No		*p*-Value
Age	12.8 ± 7.1		13.1 ± 5.9		0.431 ^a^
Time to cranioplasty (days)	171.1 ± 39.7		110.9 ± 31.5		0.005 ^a^

^a^ Fisher’s exact test; ^b^ Kruskal–Wallis test. Acute SDH: acute subdural hematoma; EDH: epidural hematoma; chronic SDH: chronic subdural hemorrhage; traumatic SAH: traumatic subarachnoid hemorrhage. We also interpreted surgical results according to six sub-groups.

## Data Availability

The original contributions presented in the study are included in the article, further inquiries can be directed to the corresponding authors.
